# Discovery of permuted and recently split transfer RNAs in Archaea

**DOI:** 10.1186/gb-2011-12-4-r38

**Published:** 2011-04-13

**Authors:** Patricia P Chan, Aaron E Cozen, Todd M Lowe

**Affiliations:** 1 Department of Biomolecular Engineering, University of California Santa Cruz, 1156 High Street, Santa Cruz, CA 95064, USA

## Abstract

**Background:**

As in eukaryotes, precursor transfer RNAs in Archaea often contain introns that are removed in tRNA maturation. Two unrelated archaeal species display unique pre-tRNA processing complexity in the form of split tRNA genes, in which two to three segments of tRNAs are transcribed from different loci, then *trans*-spliced to form a mature tRNA. Another rare type of pre-tRNA, found only in eukaryotic algae, is permuted, where the 3' half is encoded upstream of the 5' half, and must be processed to be functional.

**Results:**

Using an improved version of the gene-finding program tRNAscan-SE, comparative analyses and experimental verifications, we have now identified four novel *trans*-spliced tRNA genes, each in a different species of the Desulfurococcales branch of the Archaea: tRNA^Asp(GUC) ^in *Aeropyrum pernix *and *Thermosphaera aggregans*, and tRNA^Lys(CUU) ^in *Staphylothermus hellenicus *and *Staphylothermus marinus*. Each of these includes features surprisingly similar to previously studied split tRNAs, yet comparative genomic context analysis and phylogenetic distribution suggest several independent, relatively recent splitting events. Additionally, we identified the first examples of permuted tRNA genes in Archaea: tRNA^iMet(CAU) ^and tRNA^Tyr(GUA) ^in *Thermofilum pendens*, which appear to be permuted in the same arrangement seen previously in red alga.

**Conclusions:**

Our findings illustrate that split tRNAs are sporadically spread across a major branch of the Archaea, and that permuted tRNAs are a new shared characteristic between archaeal and eukaryotic species. The split tRNA discoveries also provide new clues to their evolutionary history, supporting hypotheses for recent acquisition via viral or other mobile elements.

## Background

Transfer RNA (tRNA) genes play an essential role in protein translation in all living cells. Among the various stages of maturation that are required to generate functional tRNAs, intron removal is a key processing event occurring in precursor tRNAs (pre-tRNAs) in eukaryotes and Archaea. While the majority of these introns are found one nucleotide downstream of the anticodon, some archaeal species have introns scattered among seemingly random, 'noncanonical' positions in tRNA genes. These noncanonical introns preserve a general bulge-helix-bulge (BHB) secondary structure that is similar to canonical introns [1], and are found almost exclusively in members of the phylum Crenarchaeota, with most species containing only a small number of noncanonical introns. The hyperthermophile *Pyrobaculum aerophilum *is an exceptional case, containing a total of 21 noncanonical introns in 46 tRNAs, more than four times as many as in any other species [1]. With the availability of four recently sequenced *Pyrobaculum *genomes (*Pyrobaculum arsenaticum*, *Pyrobaculum calidifontis*, *Pyrobaculum islandicum*, and *Thermoproteus neutrophilus*, to be reclassified as a *Pyrobaculum *species), it was found that *P. calidifontis *harbors a superlative total of over 70 tRNA introns, the vast majority being noncanonical [2,3].

A separate but potentially related class of tRNAs requiring unusual processing was first found in *Nanoarchaeum equitans*, an obligate archaeal hyperthermophilic symbiont with an extremely small genome. It contains six *trans*-spliced split tRNAs, encoded by genes that are broken into halves and distantly separated in the genome [4,5]. These *trans*-spliced tRNAs are similar to pre-tRNAs containing canonical or noncanonical introns in that they form a BHB secondary structure at the exon-splicing junction, which is likely processed by a common endonuclease [6]. Although this finding was considered an exceptional process in an unusual organism, the discovery of *trans*-spliced tRNAs in the free-living thermophilic crenarchaeon *Caldivirga maquilingensis *hints at a broader relevance [7,8]. Unfortunately, the large evolutionary distance between *N. equitans *and *C. maquilingensis*, and the lack of similarity in characteristics between their split tRNAs limit estimates of their age and the genome dynamics that might be involved.

Atypical tRNAs are not limited to archaeal species. Permuted tRNAs with a 3' half positioned upstream of the 5' half, creating a BHB-like motif when paired at their termini, were identified in the genomes of several unicellular algae, including the red alga *Cyanidioschyzon merolae *[9,10]. Various hypotheses have been proposed to explain the existence of these introns and fragmented tRNA genes, including a possible archaeal origin [8,11-14]. However, the mechanism of their acquisition and biological significance is still not known.

With the increasing number of sequenced archaeal genomes, there are opportunities to uncover new cases of exceptional tRNA encoding, which could provide important clues to understanding the evolutionary origins and mechanistic details shared among *trans*-spliced, permuted, and atypical intron-containing tRNAs. Here we report two key discoveries with new evolutionary implications. First, we describe four novel *trans*-spliced tRNAs, distributed among half (4 out of 8) of the species with decoded genomes in the Desulfurococcales branch of the Crenarchaeota. Unlike the *trans*-spliced tRNAs previously observed in *N. equitans *and *C. maquilingensis *[4,8], the genomic proximity of the newly discovered tRNA gene fragments suggests relatively recent introduction into these genomes. Second, we describe strong evidence for the first examples of permuted tRNAs in Archaea, which have striking resemblance to permuted tRNAs recently found in eukaryotic algal genomes. Our findings demonstrate that these special tRNA features are not as rare as once perceived, increasing their biological relevance as well as providing valuable additional data points for future efforts to determine their origins and required co-factors.

## Results

### Split tRNA^Asp(GUC) ^in *Aeropyrum *and *Thermosphaera *consist of adjacent halves

Our initial investigation focused on tRNAs with properties that place them at the extremes of archaeal tRNA characteristics. tRNA introns identified in sequenced archaeal genomes have sizes ranging from 11 to 129 nucleotides with a median of 15 nucleotides [2]. Besides introns of tRNA^Trp(GUC) ^that encode an embedded C/D box small RNA (sRNA) [15-17], only one tRNA gene, tRNA^Asp(GUC) ^of *Aeropyrum pernix*, contains an intron exceeding 100 nucleotides (Table S1 in Additional file 1). Using an archaeal-specific version of snoscan [18], and manual alignment to other predicted C/D box sRNAs in *A. pernix *and other archaeal species, we failed to find any trace of a C/D box sRNA that might explain the long tRNA intron. Promoter analyses revealed a strong signal in the middle of the tRNA^Asp(GUC) ^intron, including both the transcription factor B response element and TATA box, encoded on the same strand as the tRNA gene. This high-confidence promoter prediction scores better than 88% of all predicted transcripts in the genome (Figure S1 in Additional file 1), better than 63% of the predicted promoters for annotated tRNA genes in *A. pernix*, and is highly similar to the promoter upstream of the 5' end of the tRNA^Asp(GUC) ^gene (Table S2 in Additional file 1). The promoter's placement predicts a transcription start site 20 to 25 nucleotides upstream of the 3' end of the intron, consistent with production of a leader sequence of length and high G/C content that is similar to leaders found in the 3' halves of *trans*-spliced tRNA genes [5,8]

**Figure 1:**
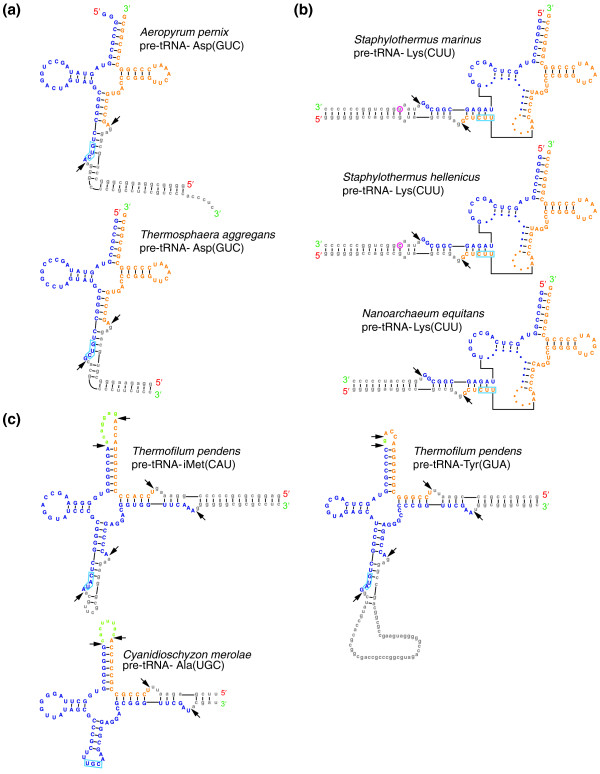
**Predicted secondary structures of *trans*-spliced and permuted precursor tRNAs**. **(a) **Mature tRNA^Asp(GUC) ^in *A. pernix *and *T. aggregans *are formed by joining the 5' half and the 3' half at position 37/38 after splicing at the bulge-helix-bulge (BHB) motif. **(b) **The 5' half and the 3' half of *trans*-spliced tRNA^Lys(CUU) ^in *Staphylothermus hellenicus *and *S. marinus marinus *join at position 30/31, same as the previously identified split tRNA^Lys(CUU) ^in *N. equitans *[5]. **(c) **Circularized permuted tRNA^iMet(CAU) ^and tRNA^Tyr(GUA) ^in *Thermofilum pendens *have the 3' half located upstream of the 5' half separated by intervening sequences represented in green. The two fragments join at position 59/60, same as the T-Ψ-C loop permuted tRNAs in the red alga *C. merolae *[9]. Pre-tRNA^Ala(UGC) ^in *C. merolae *is shown for comparison. The 5' half of tRNA transcripts are represented in blue, the 3' halves in orange. Black arrows indicate positions of splicing. Anticodons are boxed in light blue.

**Figure 2:**
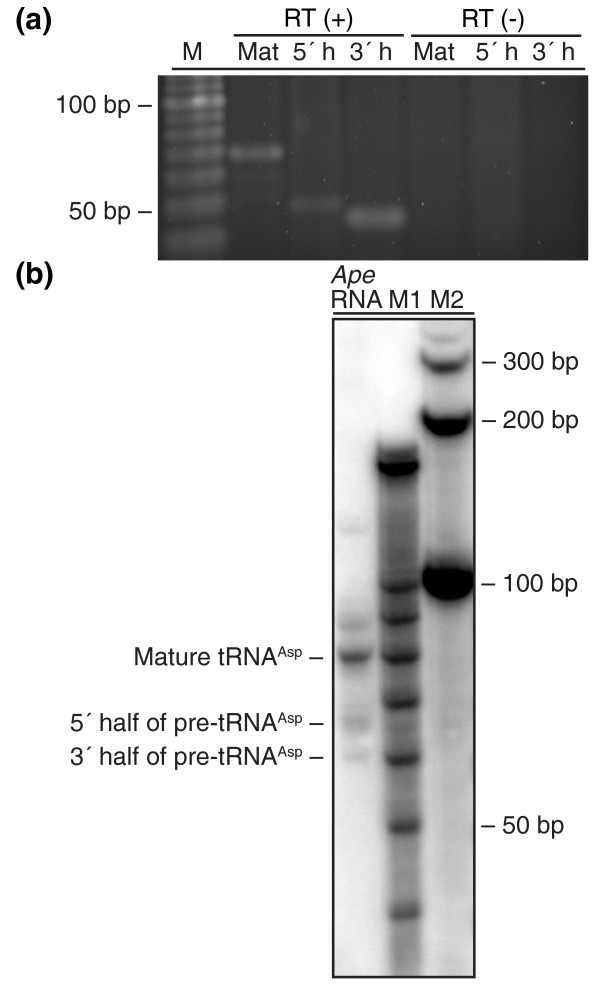
**RT-PCR and northern analysis of tRNA^Asp(GUC) ^in *A. pernix***. **(a) **Expression analysis of mature tRNA^Asp(GUC) ^(Mat), 5' half of pre-tRNA^Asp(GUC) ^(5' h), and 3' half of pre-tRNA^Asp(GUC) ^(3' h) using RT-PCR. M represents the 10-bp DNA ladder. The band sizes correspond to the sizes of the PCR products based on selected primers, but not the transcript sizes of the mature tRNA and the halves. Negative controls without reverse transcriptase (RT (-)) displayed no PCR products in comparison. **(b) **Northern analysis of tRNA^Asp(GUC) ^using radiolabeled DNA probe that spans the mature tRNA^Asp(GUC) ^and the region between the two fragments. The mature tRNA, 5' half transcript and the 3' half transcript are as marked. No expression was found corresponding to the 199-nucleotide transcript originally predicted as pre-tRNA^Asp(GUC) ^with a 121-nucleotide intron. Bands at approximately 90 nucleotides and 125 nucleotides are expected due to cross-hybridization of highly similar tRNA sequences in other tRNA transcripts. M1 and M2 represent the 10-bp and 100-bp RNA ladders, respectively.

With the aid of an improved version of tRNAscan-SE [19,20], we found that tRNA^Asp(GUC) ^can be modeled as a combination of two separate transcripts joined between position 37 and 38, the canonical intron position (Figure 1a, Table 1). Similar to other split tRNAs, a canonical BHB (helix-bulge-helix-bulge-helix - hBHBh') secondary structure at the exon-splicing junction is observed, followed by a 14-bp G/C-rich stem. RT-PCR with transcript-specific primers and sequencing of PCR-derived clones verify expression of the mature tRNA and the two tRNA halves (Figure 2a). To further confirm that the two halves are separate transcripts, we conducted northern analysis with specific probes antisense to the two halves and the full-length 199-nucleotide precursor tRNA. Results show that the two pre-tRNA halves are present at their expected sizes, as is the predicted mature tRNA (Figure 2b). Interestingly, an amplified RT-PCR product corresponding to the size of a 199-nucleotide pre-tRNA^Asp(GUC) ^was observed, but its absence in the northern analysis suggests this is a very low abundance read-through transcript that may be spliced, but does not contribute significantly to the mature tRNA abundance. Thus, this tRNA may be a true evolutionary intermediate between the intron-spliced and *trans*-spliced forms.

**Table 1:** Summary of *trans*-spliced and permuted tRNAs

			5' half of tRNA		3' half of tRNA			
					
Organism	tRNA isotype	Anticodon	5' Start	Size (bp) Pre/Mature	5' Start	Size (bp) Pre/Mature	Ligation site	Intron position
*Aeropyrum pernix	Asp	GUC	372080 (+)	~67/40	372241 (+)	~58/35	37/38	
*Thermosphaera aggregans	Asp	GUC	41001 (+)	~53/40	41160 (-)	~53/35	37/38	
*Staphylothermus hellenicus	Lys	CUU	1095046 (+)	~48/31	1090266 (+)	~65/43	30/31	
*Staphylothermus marinus	Lys	CUU	1279876 (-)	~48/31	1284654 (-)	~65/43	30/31	
*Nanoarchaeum equitans*	Lys	CUU	308963 (+)	~44/32	380370 (-)	~60/43	30/31	
	Gln	UUG	419223 (-)	~49/33	356559 (+)	~61/40	32/33	
	Glu	CUC	331758 (-)	~56/40	487064 (-)	~56/35	37/38	
	Glu	UUC	436673 (-)	~56/40	487064 (-)	~5635	37/38	
	His	GUG	221794 (+)	~49/37	409246 (+)	~53/36	37/38	
	iMet	CAU	35249 (+)	~53/39	3254 (+)	~5236	37/38	
*Thermofilum pendens	iMet	CAU	539254 (-)	~89/75	539278 (-)	~43/17	59/60	37/38
	Tyr	GUA	892743 (+)	~128/117	892725 (+)	~34/17	59/60	37/38
*Cyanidioschyzon merolae*	Ala	UGC	chr6:137501 (-)	~63/58	chr6:137523 (-)	~32/14	59/60	
	Arg	CCU	chr10:636110 (+)	~88/85	chr10:636073 (+)	~47/14	59/60	14/15
	Lys	UUU	chr16:809263 (-)	~60/57	chr16:809315 (-)	~70/14	59/60	

The novel *trans*-spliced and permuted tRNAs identified in this study are marked by asterisks. The previously identified split tRNAs in *N. equitans *[4,5] and permuted tRNAs in *C. merolae *[9] are listed for comparison. The 5' start value represents the coordinates of the 5' start site of the mature tRNA. Pre, pre-tRNA; Mature, mature tRNA.

**Figure 3:**
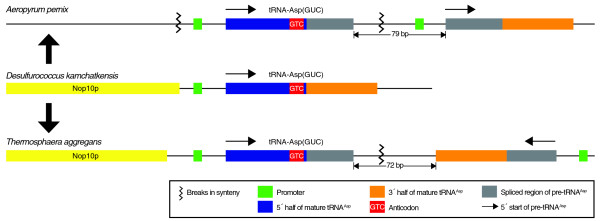
**Proposed evolutionary relationship between tRNA^Asp(GUC) ^in *D. kamchatkensis*, *A. pernix*, and *T. aggregans***. *D. kamchatkensis *has a typical linear tRNA^Asp(GUC) ^which represents the likely ancestor of the split tRNA^Asp(GUC) ^genes in *A. pernix *and *T. aggregans*. The 5' and 3' halves of pre-tRNA^Asp(GUC) ^are located adjacent to each other in *A. pernix *and *T. aggregans*. The two halves in *A. pernix *are transcribed on the forward strand while those in *T. aggregans *are transcribed on opposite strands. Breaks in synteny were observed between the tRNA halves and upstream of the 5' half in *A. pernix*.

tRNA^Asp(GUC) ^in *A. pernix *is the first example of a *trans*-spliced tRNA encoded by separate, but directly adjacent transcripts. Upon re-examination of all tRNAs in Archaea for a similar pattern, we found that the tRNA^Asp(GUC) ^ortholog in *Thermosphaera aggregans*, a crenarchaeon in the same phylogenetic order Desulfurococcales, also appears to be split with the two halves joined at the canonical intron position (Figure 1a, Table 1). Similar to the arrangement in *A. pernix*, the 5' half of the *T. aggregans *split tRNA is located adjacent to the 3' half. Unlike *A. pernix*, the two halves are encoded on opposite strands in a convergently transcribed orientation (Figure 3).

**Figure 4:**
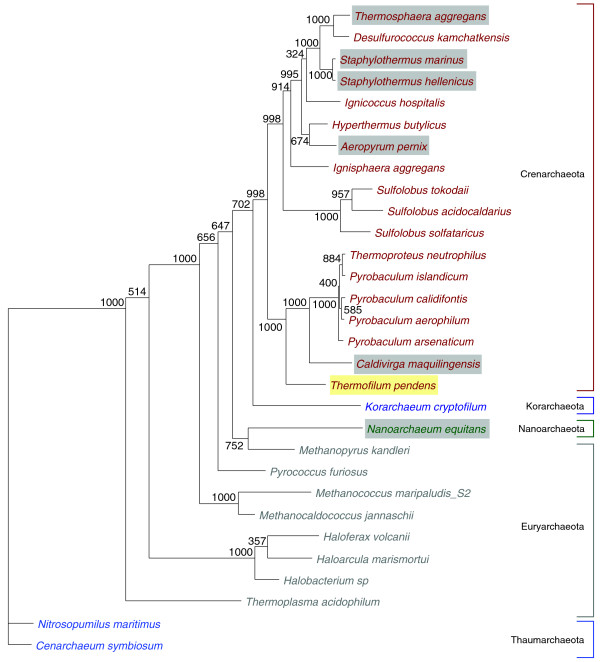
**Phylogenetic distribution of *trans*-spliced and permuted tRNAs in Archaea**. *Trans*-spliced tRNAs were identified in *A. pernix*, *T. aggregans*, *S. marinus*, and *S. marinus *in this study, in *N. equitans *by Randau and colleagues [4,5], and in *C. maquilingensis *by Fujishima and colleagues [8] (highlighted in gray). Permuted tRNAs were found in *T. pendens *in this study (yellow), and are most similar to the permuted tRNAs found in *Cyanidioschyzon merolae*, a eukaryotic algal species that is not included in the tree but would be a distant outgroup to the archaea shown here. The phylogenetic tree was generated based on the concatenation of 23S and 16S ribosomal RNAs. Sequences were aligned using ClustalW [48]. Alignments were manually adjusted using Jalview [49] to remove introns. The maximum likelihood tree was computed using PhyML [50] with general time-reversible model of sequence evolution. Numbers at nodes represent non-parametric bootstrap values computed by PhyML [50] with 1,000 replications of the original dataset.

To get an evolutionary perspective on the lineage of this tRNA gene, we examined the syntenic region in *Desulfurococcus kamchatkensis*, the closest sequenced relative to *T. aggregans*. In both of these species, a sequence of three genes is present: eIF-2A, Nop10p, then tRNA^Asp(GUC) ^(Figure 3). However, the end of the syntenic region occurs in the middle of the tRNA^Asp(GUC)^, where apparently the ancestral, uninterrupted form of tRNA^Asp(GUC) ^observed in *D. kamchatkensis *has been split and inverted in *T. aggregans*, by an unknown series of genome re-arrangement events. Examination of the tRNA^Asp(GUC) ^genomic regions for syntenic blocks in the eight sequenced Desulfurococcales species showed that every species had genome rearrangements downstream of tRNA^Asp(GUC)^, relative to every other species, and three had rearrangements upstream as well. The split tRNA^Asp(GUC)^, along with many other tRNAs [21,22], appear to be common positions for genome recombination and/or viral integration. Although *A. pernix *and *T. aggregans *are both Desulfurococcales, they are separated by four other species that are more closely related, but lack the split tRNA^Asp(GUC)^. Given that none of the other six Desulfurococcales species have a split tRNA^Asp(GUC)^, the most parsimonious explanation for these observations is that a tRNA-splitting event happened relatively recently in two independent instances among the Desulfurococcales (Figure 4). The agent causing the splitting event could be specific for these tRNA sequences, as there are only four nucleotide changes between the *A. pernix *and *T. aggregans *tRNA sequences. Notably, there are many more changes in length and sequence identity (20 nucleotide differences) in the complementary region required to join the split halves, thus disfavoring the possibility of recent lateral transfer of a precursor split tRNA gene. As such, these are likely to be the first compelling examples of 'late' acquisition of split tRNA genes based on proximal tRNA halves. Late acquisition has also been suggested recently based on comparative analyses of non-canonical tRNA introns [23].

### tRNA^Lys(CUU) ^in *Staphylothermus *resembles its ortholog in *Nanoarchaeum*

Using an improved version of the tRNAscan-SE computer program [19,20] (manuscript in preparation), we predicted 46 tRNAs in *Staphylothermus marinus*, including a missing tRNA^Lys(CUU) ^gene and an extra, low-scoring tRNA^Leu(CAA) ^prediction (26.23 bits versus all other archaeal tRNAs score >30.0 bits). This suggested a potential tRNA isotype mis-assignment. Indeed, closer examination of the low-scoring tRNA^Leu(CAA) ^revealed an almost exact match to tRNA^Lys(UUU) ^from position 30 to the 3' terminus, with no similarity upstream of position 30. The single difference between these two sequences is a U to C change, aligned to anticodon position 34 in tRNA^Lys(UUU)^, consistent with the fragment being the 3' end of the missing tRNA^Lys(CUU)^. Additional sequence similarity searches identified a candidate 5' half fragment of the tRNA^Lys(CUU) ^encoded 4,500 nucleotides away (Table 1; Figure S2a in Additional file 1), ruling out the possibility of an extremely long polycistronic intron. Strong promoters matching the promoter consensus (better than 40% of other identified tRNA promoters) were found at the expected distance upstream of both candidate loci.

Intriguingly, the two halves of the candidate split tRNA^Lys(CUU) ^in *S. marinus *join between position 30 and 31, precisely the same position as in the split tRNA^Lys(CUU) ^of distantly related *N. equitans *(Table 1) [5]. A canonical BHB (hBHBh') secondary structure at the exon-splicing junction is also observed, just as in *N. equitans *tRNA^Lys(CUU)^, followed by a perfect 13-nucleotide *trans*-pairing between the regions downstream of the 5' half and the upstream of the 3' half (Figure 1b). Fortuitously, the genome of another species in the genus *Staphylothermus*, *S. hellenicus*, was recently sequenced, enabling identification of the orthologous split tRNA^Lys(CUU)^, closely resembling the counterpart in *S. marinus *(Figure 1b, Table 1). While the sequences of 3' halves in the two species are identical, there is one nucleotide difference near the end of the 5' tRNA half. The difference conserves a base pair in the *trans*-paired region, changing the predicted G-U pairing in *S. marinus *to a G-C pair in *S. hellenicus *(Figure 1b), further suggesting selective pressure to maintain a perfect 13-nucleotide *trans *interaction.

These two closely related *Staphylothermus *species give the first case in which the issue of genome stability and split tRNAs may be examined. The neighboring genes located upstream of the 5' half (Smar_1316-Smar_1321, Shell_1133-Shell_1128), in between the 5' and 3' halves (Smar_1322 - Smar_1326, Shell_1127 - Shell_1123), and downstream of the 3' half (Smar_1327 - Smar_1332, Shell_1122 - Shell_1116) retain complete synteny between species, showing a lack of recombination or integration events in this region (Figure S2 in Additional file 1). However, tRNA genes are known to be positions of genome rearrangement due to processes including transposon and viral integration [21,24-26]. For context, we examined genome rearrangement events adjacent to the other 45 ortholog pairs of tRNAs, and found that 20 of them (44%) had a break in synteny either upstream, downstream, or on both sides of the tRNAs. Thus, the split tRNA^Lys(CUU) ^arrangement since the divergence of *S. marinus *and *S. hellenicus *has been preserved with no local recombination, and is consistent with hypotheses proposing enhanced genome stability from split tRNAs.

### Permuted tRNAs in *Thermofilum pendens *have the same structure as in red alga

We computationally screened for other atypical tRNA transcripts by aligning tRNAs and their upstream promoter regions to identify unusual spacing between candidate promoters and predicted tRNA genes. When applied to the thermophilic crenarchaeon *Thermofilum pendens*, we found that the promoters of 44 mature tRNA genes, out of a total of 46 in the genome, are located in the upstream region between 30 and 49 nucleotides relative to the 5' end of the mature tRNAs (Figure S3 in Additional file 1), explained by natural variation in the lengths of 5' leaders of pre-tRNAs. The promoters of two outliers, tRNA^iMet(CAU) ^and tRNA^Tyr(GUA)^, were found at positions -72 and -65, respectively, implying 5' leaders at least 16 nucleotides longer than all others. Similar to the initial prediction of the split tRNA in *S. marinus*, tRNA^iMet(CAU) ^and tRNA^Tyr(GUA) ^in *T. pendens *scored relatively low (26.23 and 32.76 bits, respectively, compared to >60 bits for other tRNA genes in this genome). Secondary structure analysis showed that these tRNA predictions fail to form requisite tRNA cloverleaf secondary structure at the T-Ψ-C loop and the acceptor stem.

Results from an improved version of tRNAscan-SE [19,20] showed that the original predictions of tRNA^iMet(CAU) ^and tRNA^Tyr(GUA) ^in *T. pendens *are only the 5' halves of these tRNA genes. For each tRNA, a precisely matching 3' half was found between the 5' half and its predicted promoter on the same strand, suggesting that the two fragments belong to the same transcript, using a single promoter (Figure S3 in Additional file 1). Primary and secondary structure analyses strongly support that these are circularly permuted tRNAs with a BHB motif at the exon-splicing junction, located between positions 59 and 60. Unexpectedly, the split position is precisely the same as the T-Ψ-C loop-permuted tRNAs identified in the distantly related eukaryotic alga *C. merolae *(Figure 1c) [9]. The intervening sequences between the 3' and 5' halves of tRNA^iMet(CAU) ^and tRNA^Tyr(GUA) ^in *T. pendens *are 7 nucleotides and 1 nucleotide long, respectively, in comparison to intervening sequences in known algal permuted tRNAs ranging from 5 nucleotides to 85 nucleotides. Both of the genes also include a canonical intron, indicating that permuted tRNA structure and normal intron splicing are not exclusive processes. Unlike the permuted tRNAs in eukaryotes, these two archaeal tRNAs have genomically encoded 3'-terminal CCA sequences, and the tRNA sequences are quite different from those found in red alga, ruling out a recent inter-domain transfer between algal and archaeal species. However, the sequences and proteins flanking these tRNAs share strongest similarity to species outside of the Thermoproteales, suggesting these may have been part of laterally transferred regions acquired after the divergence from other sequenced Thermoproteales. Both tRNA^iMet(CAU) ^and tRNA^Tyr(GUA) ^are single-copy genes in *T. pendens *with essential decoding functions that cannot be supplanted by other tRNAs, indicating that processing of permuted genes is an essential activity in this species.

## Discussion

RNA *trans*-splicing, a processing event that joins two separate transcripts, was first discovered in the messenger RNAs of trypanosomes [27], *Caenorhabditis elegans *[28], and more recently in human endometrial stromal cells [29]. With the surprising discovery of *trans-*spliced tRNAs encoded in the minimal genome of *N. equitans *[4], and the most recent identification in a single unrelated crenarchaeal species [8], the broader significance and phylogenetic scope of these intriguing RNAs has remained uncertain. Our discovery of four novel *trans*-spliced tRNAs and two novel permuted tRNA genes greatly expands the total number of rearranged tRNAs in the Archaea to 18, in seven different species [4,8]. All archaeal *trans*-spliced tRNAs have been found either in the crenarchaeal orders Desulfurococcales or Thermoproteales (Figure 4), or in *N. equitans*, an endosymbiont of a Desulfurococcales species [30]. A re-examination of tRNA predictions in all sequenced genomes in the Crenarchaeota did not show any additional *trans*-spliced or permuted tRNAs, although we expect many more examples as the pace of new genome sequencing accelerates [31].

Whether there are ecological or genetic characters that distinguish organisms with split or permuted tRNA genes from organisms that lack such tRNA variants is an open and interesting question. Among the seven species now identified as harboring split tRNAs, the most conspicuous ecological trait is that all are hyperthermophiles. Soon after the discovery of split tRNAs in *N. equitans *and permuted tRNAs in *C. merolae*, Randau and Söll suggested that split tRNA genes, like tRNA introns, present a strategy for preventing the integration of viral genomes or other mobile elements into otherwise conserved tRNA genes [11,32]. This is particularly relevant for tRNAs in hyperthermophiles, which must preserve a high number of strong G-C hydrogen bonds in stems in order to maintain stable secondary structure. In combination with the many other tRNA sequence identity elements needed for proper modification, aminoacylation, and decoding function, this further limits sequence diversity, making thermophile tRNAs more similar to each other than in non-thermophiles. For example, there are just seven nucleotide changes in the mature tRNA^Lys(CUU) ^between the phylogenetically distant *Nanoarchaeum *and *Staphylothermus *split tRNA orthologs (Figure 1). However, it is worth noting that many euryarchaeal hyperthermophiles (for example, *Pyrococcus *spp.) with similar constraints on tRNA G/C content do not exhibit unusual tRNA variants. Thus, the genomic or ecological factors that distinguish crenarchaeal species with many unusual tRNAs bear further investigation.

One important distinguishing feature among the major clades of Archaea involves differences in the makeup of their tRNA splicing endonuclease complexes [33-35]. In the korarchaea and almost all of the sequenced euryarchaea, the endonuclease is constituted by just one protein, as either a homodimer (α_2_) or homotetramer (α_4_) [35]. In contrast, the crenarchaea, thaumarchaea, and *N. equitans *contain two proteins that form a heterotetramer endonuclease (α_2_β_2_) [6,36-38]. Recent studies have shown that the splicing endonuclease that removes introns from pre-tRNAs in *N. equitans *and *C. maquilingensis *also processes split tRNAs [6,8]. Our discovery of new split tRNAs only in the crenarchaea strengthens a correlation between split tRNAs and the heterotetrameric form of tRNA endonuclease.

Pre-tRNAs across the archaeal phyla (Crenarchaeota, Thaumarchaeota, Nanoarchaeota, Korarchaeota and three thermophilic euryarchaeal methanogens) contain introns located at canonical as well as noncanonical positions [1-3,39]. The majority of pre-tRNAs have zero or one intron, although some include two or even three introns [2,3]. Most of these multi-intronic pre-tRNAs have been identified in *Pyrobaculum *and *Thermofilum*, contributing to the highest tRNA intron counts observed in Archaea in these genera. However, none of the five sequenced *Pyrobaculum *species have any *trans*-spliced or permuted tRNAs. *C. maquilingensis *and *N. equitans *have the most split tRNAs (six), yet each has a relatively small numbers of tRNA introns. We concur with prior suggestions that the evolutionary selective pressures to maintain intronic or split tRNAs may be similar [11]; however, the specific genetic component or environmental vector(s) necessary for acquisition or maintenance of split versus intronic tRNAs are potentially quite different. Discovery of split or permuted tRNAs in five new species allows more focused searches for requisite proteins, protein variants, or genomic properties that correspond to the species that support these types of pre-tRNA variants. For example, it is now reasonably clear that small genome size, an important feature of *N. equitans *where split tRNAs were discovered [4], is not strongly correlated with split tRNAs: *C. maquilingensis *and the four Desulfurococcales identified with split tRNAs in this study do not have exceptionally small genomes among the crenarchaea.

The relative age of tRNA introns and split tRNAs has been a matter of open debate as well. Di Giulio [40,41] proposed that split tRNAs such as those in *N. equitans *are the ancestral forms of the single-locus tRNA genes observed in most genomes. Sugahara *et al. *[3] have suggested that the conserved intron sequences observed in *Pyrobaculum *support a late, rather than early origin for tRNA introns. In this study, instances of split tRNA^Asp(GUC) ^from species in different genera, and instances of split tRNA^Lys(CUU) ^from species within the same genus suggest multiple, relatively recent splitting or lateral transfer events within the Desulfurococcales. These are fundamentally different from prior examples of split tRNAs in several respects: the split halves are adjacent or relatively close in the genome, there is just one split tRNA per genome, and an orthologous split tRNA exists to help gauge the age and stability of flanking sequences. Careful examination of the exon-splicing junctions reveals that tRNA^Lys(CUU) ^in *S. marinus *and *S. hellenicus *are ancestrally related and occur in a syntenic region of their genomes that has been stable since their divergence. Examination of the disparate exon-splicing junctions between tRNA^Asp(GUC) ^in *A. pernix *and *T. aggregans *suggests two different local genome rearrangement events, created by a viral or mobile element that targets precisely the same position in the same tRNA. Viral genes that have integrated within tRNA genes have been identified in the euryarchaeal species *Thermococcus kodakarensis *KOD1 and *Methanococcus voltae *A3 [21]. Integrated elements that overlap tRNA genes have also been found in *Sulfolobus *and *A. pernix *[22,26]. We observed six partial tRNA fragments in *A. pernix *and one in *T. aggregans *(Table S3 in Additional file 1), potentially due to other recent viral integrations or rearrangements at tRNA loci.

## Conclusions

The *trans*-spliced and permuted tRNAs identified in this study indicate that rearrangement of tRNA genes is relatively common in at least one major branch of the thermophilic crenarchaea. Interactions between pre-tRNA splicing, 5' and 3' trimming of pre-tRNAs, tRNA modification, and tRNA editing have not been thoroughly investigated, but these present multiple related processes that may modulate the ability to support split or permuted tRNAs. The discovery of the six uniquely rearranged tRNAs in the Archaea presents new opportunities to study their evolution via their genomic context and basic sequence attributes. Based on these new examples, improved methods for detection, and increasing availability of sequenced genomes, we anticipate numerous additional split or permuted tRNAs will be identified for future study. The recent genomic events leading to formation of the split tRNAs described here illustrate an active, contemporary process in tRNA evolution.

## Materials and methods

### Genomic data

Complete genomic sequences of *A. pernix*, *S. hellenicus*, *S. marinus*, *T. pendens*, and *T. aggregans *were obtained from NCBI RefSeq (accession numbers [NC_000854], [NC_014205], [NC_009033], [NC_008698], and [NC_014160]).

### tRNA gene prediction

*Trans*-spliced and permuted tRNAs were predicted using an improved version of tRNAscan-SE (manuscript in preparation) [19,20]. Archaeal tRNA-specific and BHB motif covariance models were created for similarity searching using Infernal 1.0 [42] after pre-filtering possible candidates with tRNAscan [43] and an A/B box motif detection algorithm [44]. A default cut-off score was set to 20 bits.

### Promoter identification

To generate a training set for promoter identification, potential operons were predicted genome-wide with the requirement of a minimum intergenic separation of at least 100 nucleotides (on the same strand). A 16-mer motif search of the 90 nucleotides upstream of known genes (not annotated as putative or hypothetical genes) using MEME [45] was conducted to identify the consensus promoter, including the transcription factor B response element (one to three adenosines) plus the TATA box. A position-specific scoring matrix was generated from the alignments of the MEME results after manual inspection. Each organism's position-specific scoring matrix was used to scan the 150-bp upstream region of all non-coding and protein-coding genes to identify potential promoter regions. Ten virtual genomes for each target genome were generated using a fifth-order Markov chain to retain the base frequency of the target genome, and scanned to identify the score distribution of false positives. The promoter candidates identified were filtered according to expected position [46] and a threshold *P*-value equivalent to that of the lowest-scoring known gene.

### Culture conditions for *Aeropyrum pernix*

*A. pernix K1 *was obtained from Deutsche Sammlung von Mikroorganismen und Zellkulturen (DSMZ, Braunschweig, Germany). *A. pernix *cultures were grown in TY medium (0.4% tryptone, 0.2% yeast extract, pH 7) supplemented with 3.86 mM sodium thiosulfate [47]. These cultures were incubated aerobically at 90°C and collected at mid- to late-log phase. Cell pellets were frozen in liquid N_2 _and stored at -80°C.

### Total RNA preparation

Total RNA was extracted from the frozen cell pellets using a Polytron tissue homogenizer and TRI Reagent (Sigma-Aldrich, St. Louis, MO, USA). RNA samples were treated with TURBO DNase (Ambion, Austin, TX, USA) to remove any residual DNA, re-extracted with TRI Reagent, and normalized to 1.5 μg/μl.

### Genomic DNA preparation

Cell pellets were incubated overnight in SNET lysis buffer (400 mM NaCl, 1% SDS, 20 mM Tris-Cl, 5 mM EDTA) with 400 μg/ml proteinase K at 55°C. Genomic DNA was then extracted from cell lysate using phenol:chloroform:isoamyl alcohol. DNA samples were treated with RNaseA to remove any residual RNA and re-extracted with phenol:chloroform:isoamyl alcohol.

### RT-PCR and sequencing

Total RNA from *A. pernix *was denatured at 100°C for 5 minutes and cooled on ice for 5 minutes. First strand cDNAs were synthesized from denatured total RNA using gene-specific reverse primers and Superscript III reverse transcriptase (Invitrogen, Carlsbad, CA, USA) at 65°C for 30 minutes according to the manufacturer's instructions. These cDNA templates were PCR-amplified using forward and reverse primers spanning the mature tRNAs, the 5' tRNA halves, and the 3' tRNA halves. PCR parameters for 30 cycle amplification were as follows: denaturing at 94°C for 30 seconds, annealing at 68°C for 30 seconds, and extension at 72°C for 30 seconds, using AmpliTaq DNA polymerase (Applied Biosystems, Foster City, CA, USA). Negative controls for the reactions were conducted under the same conditions concurrently, but without the addition of reverse transcriptase at the first strand cDNA synthesis step. PCR products were cloned with the pCR-2.1-TOPO cloning kit (Invitrogen). Plasmid DNA was extracted using Zyppy plasmid miniprep kit (Zymo Research, Irvine, CA, USA). DNA samples were sequenced at the University of California Berkeley DNA Sequencing Facility. Primers used for RT-PCR are as follows: forward primer for mature tRNA^Asp(GUC) ^in *A. pernix *and its 5' half, 5'-CGCGGTAGTATAGCCTGGA-3'; forward primer for 3' half of tRNA^Asp(GUC) ^in *A. pernix*, 5'-CGGGCCTGCGGAGAG-3'; reverse primer for mature tRNA^Asp(GUC) ^in *A. pernix *and its 3' half, 5'-GCGGCCGGGATTTGAAC-3'; reverse primer for 5' half of tRNA^Asp(GUC) ^in *A. pernix*, 5'-GCGGGGCCCTTGACAG-3'.

### Northern blot analysis

Five micrograms of total RNA extracted from *A. pernix *cell cultures was denatured for 3 minutes at 90°C in an equal volume of 95% formamide gel loading buffer (Ambion), resolved on an 8% polyacryamide-urea gel by electrophoresis, and blotted onto Hybond N^+ ^membrane (GE Healthcare, Piscataway, NJ, USA) by overnight electro-transfer. A 239-nucleotide sequence including the *A. pernix *tRNA^Asp(GUC) ^and the region between the two fragments was amplified by PCR using *A. pernix *genomic DNA and gene-specific primers Sn-Ape-tRNA-Asp (5'-CCCAGTGGTAAGATATGTGAACC-3') and Asn-Ape-tRNA-Asp (5'-GGCCGCGAGGATTATTG-3'). The resulting PCR product served as the template for generating single-stranded, [α-^32^P]-ATP-labeled DNA probes by linear PCR using Asn-Ape-tRNA-Asp. Hybridizations were carried out at 42°C in UltraHyb buffer (Ambion). Hybridization patterns were determined using a PhosphorImager (Molecular Dynamics, Sunnyvale, CA, USA).

## Abbreviations

BHB: bulge-helix-bulge; bp: base pair; hBHBh': helix-bulge-helix-bulge-helix; pre-tRNAs: precursor transfer RNAs; sRNA: small RNA; tRNA: transfer RNA.

## Authors' contributions

PC identified *trans*-spliced and permuted tRNA genes, purified *Aeropyrum *total RNA and genomic DNA, performed all sequencing and northern analyses, and co-wrote the manuscript. AC grew *Aeropyrum *cultures, contributed ideas for experimental verifications, and co-edited the manuscript. TL guided the research study, performed tRNA synteny and evolutionary analyses, and co-wrote the manuscript. All authors read and approved the final manuscript.

## Supplementary Material

Additional file 1 **Additional figures and tables in PDF format**. Figure S1: predicted promoter score distribution in *A. pernix*. Figure S2: tRNA^Lys(CUU) ^in **(a) ***S. marinus *and **(b) ***S. hellenicus *loci display strong synteny on the Archaeal Genome Browser [51]. Figure S3: alignment of tRNA promoters in *T. pendens*. Table S1: summary of pre-tRNA intron size in 90 archaeal genomes. Table S2: predicted promoters of tRNA genes in *A. pernix*. Click here for file
