# Supplementary material for: Discovery of permuted and recently split transfer RNAs in Archaea
Source: Genome Biol. 2011 Apr 13;12(4):R38. doi: 10.1186/gb-2011-12-4-r38 (PMC3218864; doi:10.1186/gb-2011-12-4-r38)
Supplement: Additional file 1 — Additional figures and tables in PDF format. Figure S1: predicted promoter score distribution in A. pernix. Figure S2: tRNALys(CUU) in (a) S. marinus and (b) S. hellenicus loci display strong synteny on the Archaeal Genome Browser [51]. Figure S3: alignment of tRNA promoters in T. pendens. Table S1: summary of pre-tRNA intron size in 90 archaeal genomes. Table S2: predicted promoters of tRNA genes in A. pernix. [file gb-2011-12-4-r38-S1.DOC]

**Additional Figures and Tables**

**
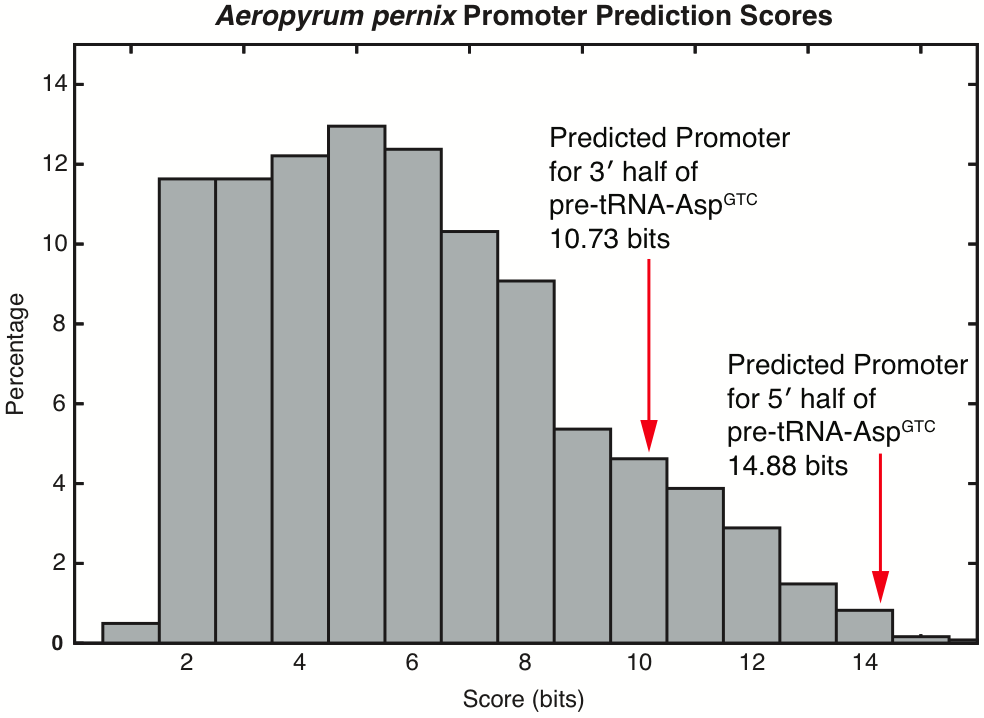
**

## Figure S1 - Predicted promoter score distribution in *A. pernix*

Histogram represents the score distribution of predicted promoters for all transcripts in the *A. pernix* genome. The scores of predicted promoters for the 5′ half and the 3′ half of pre-tRNA^Asp(GUC)^ are as marked.

**
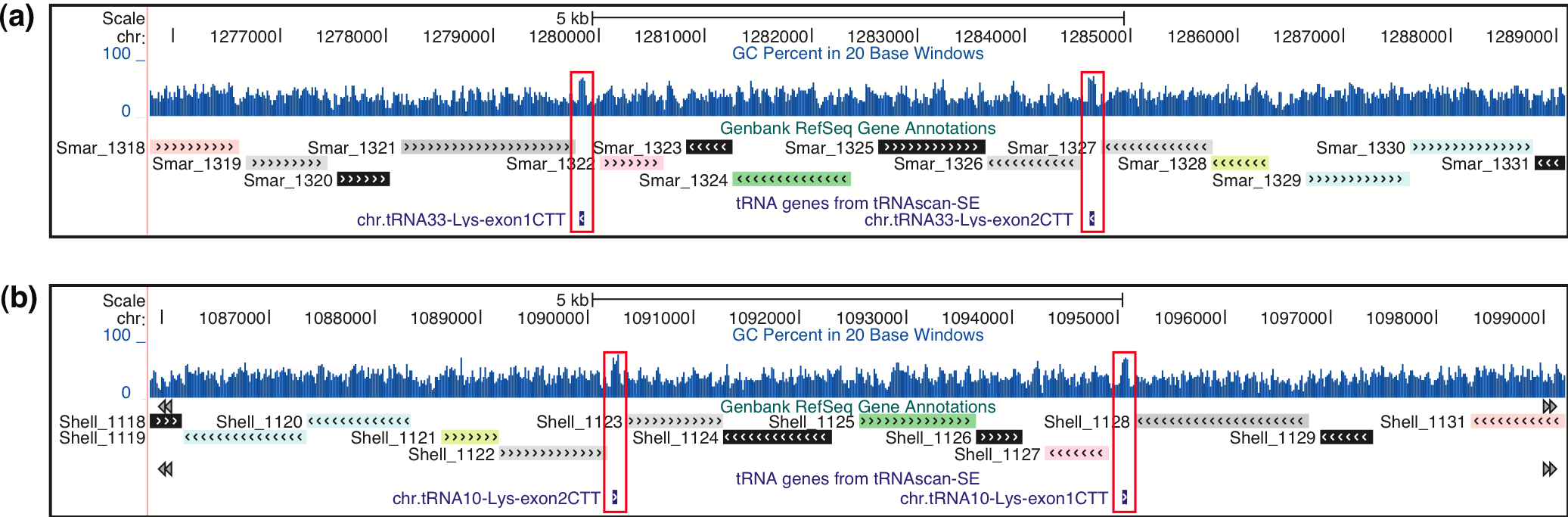
**

## Figure S2 - tRNA^Lys(CUU)^ in (a) *S. marinus* and (b) *S. hellenicus* loci display strong synteny on the Archaeal Genome Browser [50].

The blue segments located at the bottom tRNA gene track correspond to the 5′ half and the 3′ half of tRNA^Lys(CUU)^. The arrows on the genes indicate the 5′-to-3′ expression direction. The colors on the protein-coding genes represent annotations of different functional classes of clusters of orthologous groups (COG). The blue track above the genes represents the G/C content computed with a 20-base sliding window. Compared to the neighboring protein-coding genes, the two half transcripts of tRNA^Lys(CUU)^ have a high G/C content that is required for structural RNA stability in hyperthermophiles.

**
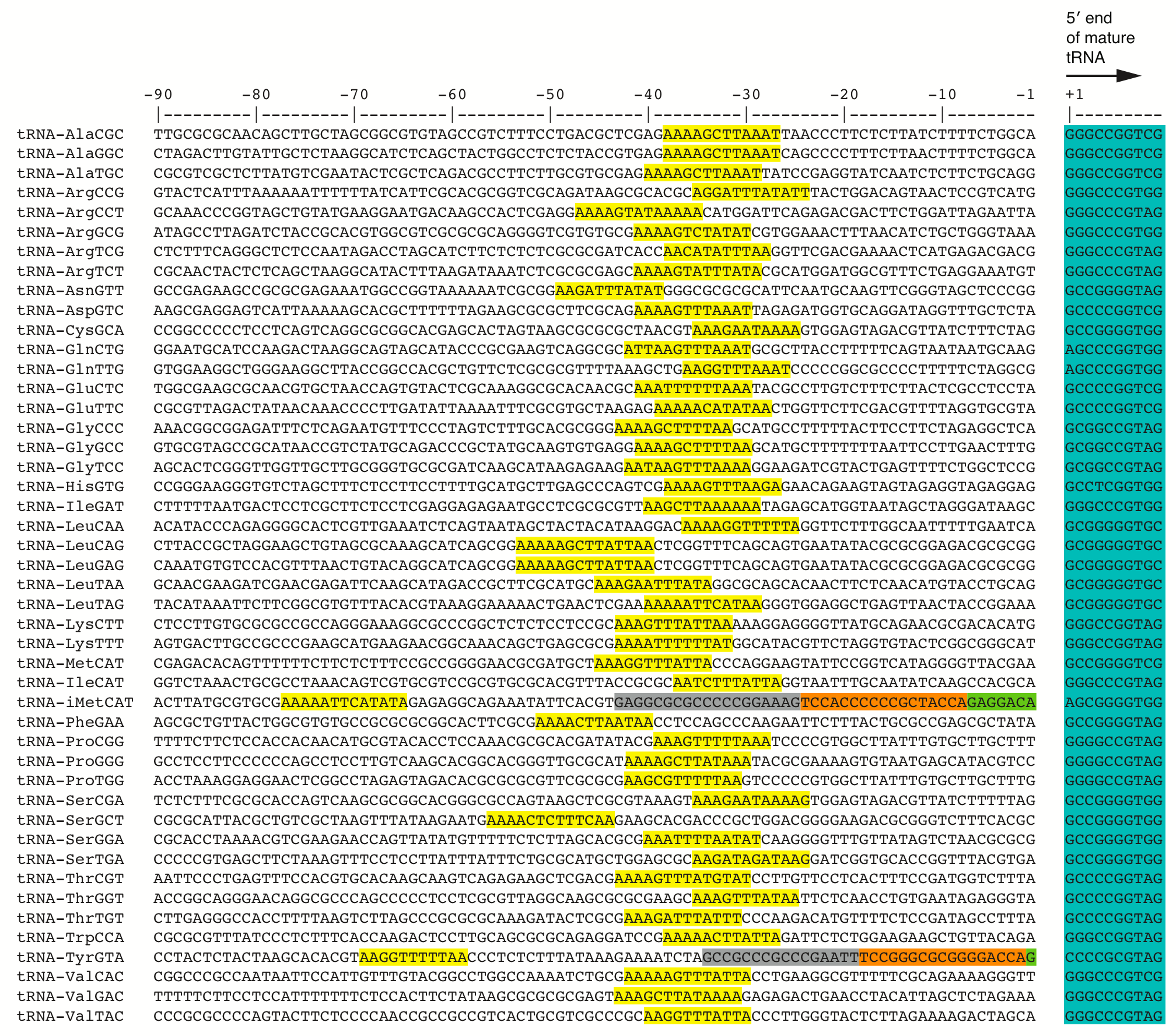
**

## Figure S3 - Alignment of tRNA promoters in *T. pendens*

The predicted promoter including BRE and TATA box of each tRNA gene is highlighted in yellow. The 5′ end of the mature tRNA-encoding sequence is highlighted in cyan. The 3′ halves of the permuted mature tRNA^iMet(CAU)^ and tRNA^Tyr(GUA)^ are highlighted in orange. Splicing regions of the 3′ half of pre-tRNAs are in grey. Intervening sequences between the 5′ and 3′ halves of the permuted tRNAs are in green. Numbering at the top indicates the position relative to the 5′ end of mature tRNAs. The black arrow shows the direction of transcription.

Table S1 - Summary of pre-tRNA intron size in 90 archaeal genomes

tRNAs and their introns were predicted using an improved version of tRNAscan-SE [19] and are publicly available at Genomic tRNA Database [2]. Red highlights the atypical predicted intron size in pre-tRNA^Asp^.

| **tRNA Isotype** | **Anticodon** | **Total Number of tRNA Genes** | **Total Number of Introns** | **Intron Length** | | |
| --- | --- | --- | --- | --- | --- | --- |
|  |  |  |  | **Minimum** | **Maximum** | **Median** |
| Trp | CCA | 88 | 95 | 13 | 129 | 65 |
| **Asp** | **GTC** | **105** | **15** | **15** | **121** | **18** |
| Tyr | GTA | 91 | 60 | 13 | 94 | 13 |
| Met | CAT | 280 | 122 | 12 | 84 | 17 |
| Pro | CGG | 73 | 26 | 15 | 79 | 18 |
| Thr | GGT | 93 | 10 | 12 | 68 | 14 |
| Ser | CGA | 70 | 28 | 13 | 61 | 24 |
| Thr | CGT | 80 | 33 | 13 | 56 | 13 |
| Glu | TTC | 106 | 37 | 14 | 55 | 15 |
| Thr | TGT | 97 | 34 | 12 | 54 | 15 |
| Arg | GCG | 91 | 11 | 13 | 52 | 16 |
| Arg | CCG | 68 | 6 | 14 | 51 | 14 |
| His | GTG | 90 | 11 | 16 | 50 | 17 |
| Ile | GAT | 94 | 24 | 12 | 49 | 12 |
| Gln | TTG | 91 | 17 | 14 | 48 | 16 |
| Pro | TGG | 90 | 19 | 15 | 48 | 18 |
| Cys | GCA | 125 | 45 | 11 | 47 | 25 |
| Gln | CTG | 78 | 16 | 15 | 47 | 16 |
| Arg | TCT | 90 | 23 | 13 | 44 | 15 |
| Ala | TGC | 121 | 12 | 13 | 40 | 15 |
| Glu | CTC | 75 | 31 | 15 | 37 | 15 |
| Pro | GGG | 88 | 39 | 13 | 37 | 21 |
| Gly | CCC | 74 | 8 | 12 | 36 | 14 |
| Leu | CAA | 75 | 19 | 12 | 36 | 15 |
| Lys | CTT | 74 | 23 | 15 | 36 | 22 |
| Lys | TTT | 98 | 19 | 14 | 36 | 23 |
| Leu | TAA | 93 | 17 | 12 | 35 | 15 |
| Asn | GTT | 95 | 30 | 11 | 34 | 14 |
| Gly | GCC | 102 | 9 | 12 | 34 | 25 |
| Ala | GGC | 90 | 4 | 14 | 33 | 14 |
| Phe | GAA | 96 | 10 | 14 | 33 | 17 |
| Ala | CGC | 76 | 7 | 15 | 32 | 16 |
| Ile | TAT | 2 | 2 | 19 | 32 | 19 |
| Val | TAC | 90 | 16 | 14 | 32 | 25 |
| Val | CAC | 82 | 9 | 16 | 31 | 18 |
| Gly | TCC | 92 | 11 | 14 | 30 | 15 |
| Ser | GGA | 93 | 9 | 12 | 29 | 14 |
| Val | GAC | 94 | 10 | 13 | 28 | 20 |
| Arg | CCT | 78 | 23 | 13 | 24 | 13 |
| Arg | TCG | 90 | 10 | 16 | 24 | 20 |
| Leu | CAG | 73 | 6 | 15 | 23 | 15 |
| Ser | TGA | 91 | 10 | 11 | 23 | 12 |
| Ser | GCT | 91 | 2 | 12 | 22 | 12 |
| Leu | GAG | 96 | 2 | 19 | 20 | 19 |
| Leu | TAG | 90 | 2 | 16 | 18 | 16 |
| Ala | AGC | 0 | 0 | 0 | 0 | 0 |
| Arg | ACG | 0 | 0 | 0 | 0 | 0 |
| Asn | ATT | 0 | 0 | 0 | 0 | 0 |
| Asp | ATC | 0 | 0 | 0 | 0 | 0 |
| Cys | ACA | 0 | 0 | 0 | 0 | 0 |
| Gly | ACC | 0 | 0 | 0 | 0 | 0 |
| His | ATG | 0 | 0 | 0 | 0 | 0 |
| Ile | AAT | 0 | 0 | 0 | 0 | 0 |
| Leu | AAG | 0 | 0 | 0 | 0 | 0 |
| Phe | AAA | 0 | 0 | 0 | 0 | 0 |
| Pro | AGG | 0 | 0 | 0 | 0 | 0 |
| Sel | TCA | 5 | 0 | 0 | 0 | 0 |
| Ser | ACT | 0 | 0 | 0 | 0 | 0 |
| Ser | AGA | 0 | 0 | 0 | 0 | 0 |
| Supres | CTA | 0 | 0 | 0 | 0 | 0 |
| Supres | TTA | 0 | 0 | 0 | 0 | 0 |
| Thr | AGT | 0 | 0 | 0 | 0 | 0 |
| Tyr | ATA | 0 | 0 | 0 | 0 | 0 |
| Val | AAC | 0 | 0 | 0 | 0 | 0 |

## Table S2 - Predicted promoters of tRNA genes in *Aeropyrum pernix*

Promoter motifs that include transcription factor B response element (BRE) and TATA-box were predicted using a genome-specific position-specific scoring matrix (see Methods). Relative position of the promoter represents the center position of the predicted promoter motif relative to the 5′ end of the mature tRNA genes (except for the 3′ half of tRNA^Asp^ which includes the 5′ leader).

| **tRNA genes** | **Promoter** | **Relative Position** | **Score (bits)** |
| --- | --- | --- | --- |
| chr.tRNA4-AspGTC-exon1 | GCAAAGCTTTAAACCC | -33 | 14.88 |
| chr.tRNA34-PheGAA | TTTAAGGTTAAAAACC | -43 | 13.85 |
| chr.tRNA48-ThrTGT | CAAACCCTTTAAACCC | -40 | 13.48 |
| chr.tRNA28-LeuGAG | CTAACACTTTATAGCC | -37 | 13.47 |
| chr.tRNA38-GlyGCC | GTAAATCTTTAACCCT | -36 | 12.29 |
| chr.tRNA39-AlaGGC | CTAATCCTTAAAACCT | -68 | 12.20 |
| chr.tRNA50-SerGCT | GTAAACTTTTATTCCC | -40 | 12.05 |
| chr.tRNA7-ValTAC | GTAAACCTATAAGACC | -43 | 12.00 |
| chr.tRNA19-GlyTCC | TTATAGGTTAAAAACC | -56 | 11.44 |
| chr.tRNA6-LeuCAA | CCTAGACTATAAAATA | -17 | 11.40 |
| chr.tRNA27-AlaTGC | TAATAGGTTAAAAACT | -39 | 11.166 |
| chr.tRNA45-ValCAC | CGTAAGCTATAAACCC | -44 | 11.05 |
| chr.tRNA25-LeuCAG | CAAAGCGTTTAAAGGC | -36 | 11.00 |
| chr.tRNA30-ProGGG | TTCAAACTTTTTACCC | -39 | 10.87 |
| chr.tRNA10-IleGAT | TCTAGACTTAATAAGC | -40 | 10.86 |
| chr.tRNA29-SerGGA | GTAGATCTTTATAACG | -34 | 10.75 |
| chr.tRNA4-AspGTC-exon2 | GCAAACGTTTTTAACC | -32 | 10.73 |
| chr.tRNA32-GlyCCC | GCTAACCTTTAAGCCC | -37 | 10.68 |
| chr.tRNA42-LeuTAG | TAAACCGTTTTAACCC | -37 | 10.55 |
| chr.tRNA5-IleCAT | GAAACAGTATTAAACC | -42 | 10.43 |
| chr.tRNA52-ThrCGT | GATAACCCTTAAACCC | -41 | 9.53 |
| chr.tRNA12-ArgTCT | TCCATACTATAAAGGC | -39 | 9.50 |
| chr.tRNA13-iMetCAT | ATGACACTATAATACT | -24 | 9.24 |
| chr.tRNA14-GluCTC | GAAAGACTCAAAAACC | -72 | 8.91 |
| chr.tRNA16-ValGAC | GGAAACCTATAAGACC | -43 | 8.87 |
| chr.tRNA24-GluTTC | TCTAACCCTTAAAGCC | -36 | 8.45 |
| chr.tRNA22-HisGTG | CTAGATCTATATAGGT | -138 | 8.20 |
| chr.tRNA3-AlaCGC | GCAAGGCTTATTATCC | -42 | 8.06 |
| chr.tRNA20-GlnCTG | GCACACCTATTTAACC | -35 | 7.94 |
| chr.tRNA23-CysGCA | GTGAACCTCTAAAACC | -35 | 7.92 |
| chr.tRNA51-ProCGG | AGAAGCCTTTAAGCCT | -57 | 7.77 |
| chr.tRNA9-ArgGCG | GAACCACTATAACCAC | -33 | 7.72 |
| chr.tRNA47-MetCAT | ACCAAGCTTAACACAC | -46 | 7.43 |
| chr.tRNA31-ThrGGT | CTAACCAATTAAACCC | -41 | 7.34 |
| chr.tRNA49-LysCTT | GAAAACTCTTATAGCC | -41 | 7.31 |
| chr.tRNA15-LysTTT | AATAACCTTATGAGAG | -18 | 7.31 |
| chr.tRNA26-ArgCCT | GTAACGCTTTTAGCCA | -55 | 7.18 |
| chr.tRNA41-AsnGTT | GAAACCGTTAAAGCCA | -35 | 7.11 |
| chr.tRNA33-GlnTTG | AATATCCTTTAGGGCA | -43 | 6.95 |
| chr.tRNA43-SerCGA | CCAAGGGATTAAAACC | -54 | 6.74 |
| chr.tRNA1-ArgCCG | GAGACGATAAAAATTC | -33 | 6.73 |
| chr.tRNA21-TyrGTA | TTCAAACCTTATAGGT | -35 | 6.62 |
| chr.tRNA35-SerTGA | GAGACTCTATTAACTC | -37 | 6.21 |
| chr.tRNA40-TrpCCA | TTAGCGATTTTAAGCC | -42 | 6.01 |
| chr.tRNA17-ArgTCG | GAAAACCATAACACAC | -34 | 5.53 |
| chr.tRNA36-LeuTAA | GCGAGTATTTTTAGCC | -38 | 3.70 |
| chr.tRNA44-ProTGG | Not available (polycistronic) |  |  |

## Table S3 - Partial predicted archaeal tRNAs

tRNA fragments were predicted by tRNAscan-SE [19] and verified by manual sequence alignments for tRNA isotype identification.

| **Organism** | **Partial tRNA coordinates** | **tRNA isotype** | **Anticodon** | **Consensus tRNA positions** |
| --- | --- | --- | --- | --- |
| *Aeropyrum pernix* | 200292-200336 (+) | Arg | CCG | 32-76 |
|  | 1666802-1666846 (+) | Arg | CCG | 32-76 |
|  | 478150-478213 (-) | Leu | TAA | 21-76 |
|  | 898974-899036 (+) | Thr | TGT | 7-69 |
|  | 328963-329007 (-) | Val | CAC | 32-76 |
|  | 580684-580733 (+) | Val | TAC | 27-76 |
| *Thermosphaera aggregans* | 843106-843141 (+) | Ser | TGA | 30-55 |
